# Prenatal Air Pollution and Reduced Birth Weight: Decline in Placental Mitochondria as a Potential Mechanism

**DOI:** 10.1289/ehp.124-A98

**Published:** 2016-05-01

**Authors:** Julia R. Barrett

**Affiliations:** Julia R. Barrett, MS, ELS, a Madison, WI–based science writer and editor, is a member of the National Association of Science Writers and the Board of Editors in the Life Sciences.

Strong epidemiological evidence links prenatal exposure to ambient air pollution and outcomes including low birth weight, intrauterine growth restriction, and preterm birth.^1^^,^^2^ A new study finds evidence that the association between prenatal air pollution exposure and reduced birth weight may be mediated in part by a decline in the mitochondrial content of the placenta.^3^

During pregnancy, the placenta supports the nourishment, growth, and development of the fetus, and mitochondria within the cells of the placenta are essential to these processes.^4^ Mitochondria, the cellular organelles that regulate energy production, are easily damaged by reactive oxygen species generated by oxidative stress. Mitochondrial DNA (mtDNA) is inherently more vulnerable to oxidative stress than nuclear DNA because it lacks protective and repair mechanisms.^4^ Such damage could ultimately lead to diminished numbers of mitochondria and impaired energy flow, which would undermine cellular functions.^5^

**Figure:**
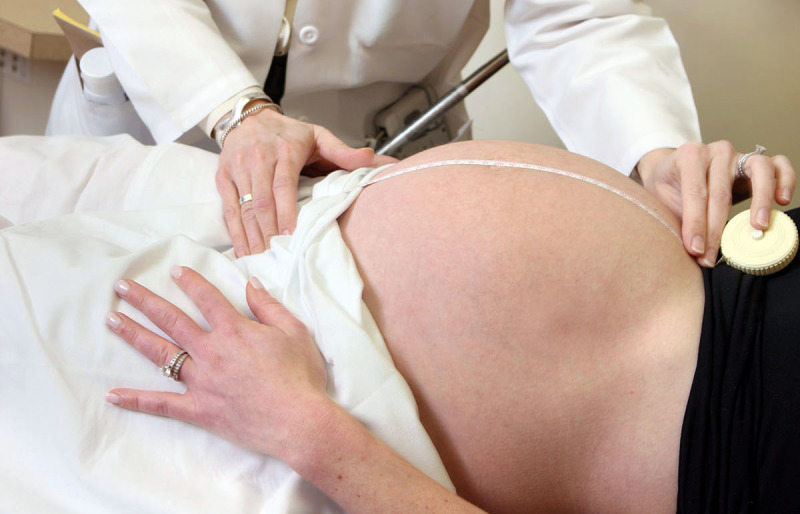
Low birth weight, intrauterine growth restriction, and preterm birth are all strongly associated with exposure to air pollution. A reduction in placental levels of mitochondrial DNA could help explain why. © Katrina Wittkamp/Getty Images

Air pollution is known to induce oxidative stress; thus, it could adversely affect placental mitochondria and thereby impair the ability of the placenta to support the growing fetus.^3^^,^^5^ A potential consequence of placental impairment is low birth weight, which in turn is associated with other detrimental health outcomes in children, both short- and long-term.^2^

The current study draws on data collected through two independent birth cohort studies conducted in Europe: Infancia y Medio Ambiente (INMA) in Spain, and Environmental Influence on Ageing (ENVIR*ON*AGE) in Belgium. Demographic data and information on smoking status, residence location, and pre-pregnancy body mass index were obtained for all participants, and birth records were used to gather information on newborns’ sex, birth weight, gestational age, and delivery. For one-quarter of the INMA participants and all ENVIR*ON*AGE participants, placentas were collected at birth, and their nuclear DNA and mtDNA content was measured.

Ambient air pollution exposure for both cohorts was represented by nitrogen dioxide (NO_2_) concentrations. Exposures were averaged for each trimester and for the whole pregnancy.

The current statistical analyses focused on 376 INMA participants and 550 ENVIR*ON*AGE participants. The researchers found that prenatal NO_2_ exposures overall and for each trimester were inversely associated with both placental mtDNA content and birth weight. Specifically, each 10-μg/m^3^ increase in NO_2_ over the course of pregnancy was associated with an average 48-g reduction in birth weight across both cohorts.^3^

The association between NO_2_ and birth weight was stronger in the INMA cohort than in the ENVIR*ON*AGE cohort (an average 66-g versus 20-g reduction), which could be explained by differences in factors such as exposure assessment methods, population characteristics, and study design.^3^ For instance, for the INMA cohort, NO_2_ was measured by outdoor passive samplers distributed throughout the study areas, with land use regression models accounting for residential location. The NO_2_ exposure estimates in the ENVIR*ON*AGE cohort depended on residential address, satellite data, and a dispersion model that incorporated ground-based data.

Prenatal NO_2_ exposures were also inversely associated with placental mtDNA content, and placental mtDNA and birth weight were positively correlated. Based on these results, the authors concluded that placental mtDNA content may mediate the relationship between prenatal NO_2_ exposure and birth weight.^3^

“We saw very clearly that the mtDNA was indeed related to birth weight,” says lead investigator Tim Nawrot, an assistant professor of environmental epidemiology at Hasselt University in Belgium. “Babies with a lower mtDNA content in the placenta showed a lower birth weight in both cohorts.” The authors point to the lack of data on indoor air exposures—which also have been associated with changes in birth weight—as a limitation of the study.

“Overall, I see this study as an important piece of the puzzle as far as the weight of evidence on air pollution and pregnancy outcomes,” says David Stieb, a public health physician and epidemiologist at Health Canada, who was not involved in the study. “There have been dozens of epidemiological studies looking at these associations, and there does seem to be a fairly consistent signal now, especially for air pollution and reduced birth weight.” With limited evidence for the mechanisms that could explain that association, “this study could definitely help fill the gap,” says Stieb.

The results will need to be confirmed through further epidemiological investigations, long-term prospective studies, and experimental research. “We will certainly look at whether the mitochondrial function in the placenta is related to future aspects of development,” says Nawrot. “What we want to establish is whether the mitochondrial-related weight reduction in early life is related to disease development later in life.”
